# Visualisation of time-varying respiratory system elastance in experimental ARDS animal models

**DOI:** 10.1186/1471-2466-14-33

**Published:** 2014-03-02

**Authors:** Erwin J van Drunen, Yeong Shiong Chiew, Christopher Pretty, Geoffrey M Shaw, Bernard Lambermont, Nathalie Janssen, J Geoffrey Chase, Thomas Desaive

**Affiliations:** 1 University of Canterbury, Christchurch 8041, New Zealand; 2 University of Liège, Liège, Belgium; 3 Christchurch Hospital, Christchurch 8011, New Zealand; 4 University Hospital of Liège, Liège, Belgium

**Keywords:** Mechanical ventilation, PEEP, Time-varying elastance, Model-based methods, ARDS, Monitoring

## Abstract

**Background:**

Patients with acute respiratory distress syndrome (ARDS) risk lung collapse, severely altering the breath-to-breath respiratory mechanics. Model-based estimation of respiratory mechanics characterising patient-specific condition and response to treatment may be used to guide mechanical ventilation (MV). This study presents a model-based approach to monitor time-varying patient-ventilator interaction to guide positive end expiratory pressure (PEEP) selection.

**Methods:**

The single compartment lung model was extended to monitor dynamic time-varying respiratory system elastance, *E*_*drs*_, within each breathing cycle. Two separate animal models were considered, each consisting of three fully sedated pure pietrain piglets (oleic acid ARDS and lavage ARDS). A staircase recruitment manoeuvre was performed on all six subjects after ARDS was induced. The *E*_*drs*_ was mapped across each breathing cycle for each subject.

**Results:**

Six time-varying, breath-specific *E*_*drs*_ maps were generated, one for each subject. Each *E*_*drs*_ map shows the subject-specific response to mechanical ventilation (MV), indicating the need for a model-based approach to guide MV. This method of visualisation provides high resolution insight into the time-varying respiratory mechanics to aid clinical decision making. Using the *E*_*drs*_ maps, minimal time-varying elastance was identified, which can be used to select optimal PEEP.

**Conclusions:**

Real-time continuous monitoring of in-breath mechanics provides further insight into lung physiology. Therefore, there is potential for this new monitoring method to aid clinicians in guiding MV treatment. These are the first such maps generated and they thus show unique results in high resolution. The model is limited to a constant respiratory resistance throughout inspiration which may not be valid in some cases. However, trends match clinical expectation and the results highlight both the subject-specificity of the model, as well as significant inter-subject variability.

## Background

Acute respiratory distress syndrome (ARDS) [1] results in a stiffer lung [2]. ARDS patients are admitted to the intensive care unit (ICU) and require mechanical ventilation (MV) for breathing support. Positive end expiratory pressure (PEEP) is applied to aid recovery by improving gas exchange and maintaining recruited lung volume [3-6]. However, variation in a patient’s response to MV and the heterogeneity of ARDS means there is a need to determine optimal patient-specific PEEP [3,7].

ARDS involves alterations in a patient’s breath-to-breath respiratory mechanics. Modelling these alterations can potentially provide a non-invasive, patient-specific method to obtain clinically and physiologically useful information to guide treatment in real-time [8-11]. This approach can provide unique insight into disease progression and patient response to MV [12-15]. However, real-time monitoring of respiratory mechanics throughout MV treatment is, to date, limited in clinical application and impact [16].

Dynamic respiratory system elastance (*E*_*drs*_) is a breath-specific time-varying lung elastance [17]. Dynamic elastance within a breath provides unique insight into a patient’s breathing pattern, revealing lung recruitment and overdistension [17,18]. In addition, identifying when minimum *E*_*drs*_ (maximum compliance) occurs during PEEP titration can help identify an optimal patient-specific PEEP to minimise work of breathing (WOB) and maximise recruitment without inducing further lung injury [19,20]. This work presents a novel method of visualising the time-varying respiratory elastance to provide a higher resolution metric to guide MV therapy.

## Methods

### Dynamic respiratory system elastance model

The equation of motion describing the airway pressure as a function of the resistive and elastic components of the respiratory system is defined as [21]:

(1) $$P_{\mathrm{aw}}\left(t\right)=R_{\mathrm{rs}}\times Q\left(t\right)+E_{\mathrm{rs}}\times V\left(t\right)+P_{0}$$

where *P*_*aw*_ is the airway pressure, *t* is time, *R*_*rs*_ is the series resistance of the conducting airway, *Q* is the air flow, *E*_*rs*_ is an overall respiratory system elastance (1/compliance), *V* is the lung volume and *P*_*0*_ is the offset pressure.

During inspiration, a fully sedated patient will have a near constant chest wall elastance, *E*_*cw*_. Thus, changes in the respiratory system elastance, *E*_*rs*_, are attributed directly to the patient’s lung elastance, *E*_*lung*_, as shown in Equation 2, thereby providing insight into patient condition and ARDS severity [2,21].

(2) $$E_{\mathrm{rs}}=E_{\mathrm{cw}}+E_{\mathrm{lung}}$$

Equation 3 describes an integral-based method [22] used to estimate values of *E*_*rs*_ and *R*_*rs*_ that best fit Equation 1. Integral-based parameter identification is similar to multiple linear regression, where using integrals significantly increases robustness to noise [17,22].

(3) $$\int P_{\mathrm{aw}}\left(t\right)\mathrm{dt}=R_{\mathrm{rsIB}}\times \int Q\left(t\right)\mathrm{dt}+E_{\mathrm{rsIB}}\times \int V\left(t\right)\mathrm{dt}+\int P_{0}\mathrm{dt}$$

Respiratory resistance is assumed constant throughout a breath [17], but can vary with PEEP and time due to opening or closing of respiratory system airways [12,14,17,23]. Thus, once *R*_*rs*_ is determined for a particular breath using Equation 3, it is substituted into Equation 4 where dynamic lung elastance, *E*_*drs*_, is defined as a time-varying lung elastance, such that *E*_*rs*_ is effectively the average of *E*_*drs*_.

(4) $$P_{\mathrm{aw}}\left(t\right)=R_{\mathrm{rs}}\times Q\left(t\right)+E_{\mathrm{drs}}\left(t\right)\times V\left(t\right)+P_{0}$$

Thus, *E*_*drs*_ can be determined from:

(5) $$E_{\mathrm{drs}}\left(t\right)=\frac{P_{\mathrm{aw}}\left(t\right)-P_{0}-R_{\mathrm{rs}}\times Q\left(t\right)}{V\left(t\right)}$$

In this way, significantly more insight is gained into the respiratory elastance over the course of inspiration than can be provided by a single value of *E*_*rs*_.

During a PEEP increase, recruitment of new lung volume outweighs lung stretching provided that the global measure of *E*_*drs*_ decreases breath-to-breath [17]. Hence, the dynamic trajectory of *E*_*drs*_ captures the overall balance of volume (recruitment) and pressure (risk) within the lung.

### Experimental data

Two experimental ARDS animal models are considered, each using three fully sedated pure pietrain piglets. The criterion for ARDS is limited to hypoxemia monitoring where the PaO_2_/FiO_2_ (PF ratio) is less than 300 mmHg.

1. **Oleic Acid ARDS Models**[24]: Each subject (Subjects 1–3) was sedated and ventilated through a tracheotomy under volume control (tidal volume, *V*_*t*_ = 8–10 ml/kg) with an inspired oxygen fraction (FiO_2_) of 0.5 and a respiratory rate of 20 breaths/min using an Engström CareStation ventilator (Datex, General Electric, Finland). ARDS was induced using oleic acid [25] and the arterial blood gas (ABG) was monitored half hourly. Once diagnosed with ARDS, each subject underwent a staircase recruitment manoeuvre (RM) with a PEEP level sequence of 5 – 10 – 15 – 20 – 15 – 10 – 5 cmH_2_O [26]. Breathing was maintained for approximately 10–15 breathing cycles at each PEEP level. Airway pressure and flow data were acquired using the Eview module provided with the ventilator. The data sampling rate was 25 Hz.

2. **Lavage ARDS Models**: After sedation and intubation via tracheotomy, the piglets (Subjects 4–6) were ventilated by intermittent positive pressure ventilation mode using a Drager Evita2 ventilator (Drager, Lubeck Germany). The ventilator was set to deliver a tidal volume of 8–10 ml/kg with a FiO_2_ of 0.5 at a respiratory rate of 20 breaths/min. Each subject underwent surfactant depletion using lavage methods [25]. The ABG was monitored and once diagnosed with ARDS, each subject underwent a staircase RM with PEEP settings at 1 – 5 – 10 – 15 – 20 – 15 – 10 – 5 – 1 mbar [26]. Breathing was maintained for approximately 10–15 breathing cycles at each PEEP level. Airway pressure and flow were measured using a 4700B pneumotachometer (Hans Rudolph Inc., Shawnee, KS) at a sampling rate of 200 Hz. Calibration was performed by matching pressure flow curve from the pneumotachometer to the peak inspiratory pressure (PIP), positive end expiratory pressure (PEEP), flow displayed in Drager Evita2 ventilator (Drager, Lubeck, Germany).

Airway pressure and flow rate data was analysed using MATLAB (The Mathworks, Natick, Massachusetts, USA). All experimental procedures, protocols and the use of data in this study were reviewed and approved by the Ethics Committee of the University of Liege Medical Faculty.

### Visualisation of the dynamics (Elastance)

Dynamic respiratory system elastance (*E*_*drs*_) varies within a breath as recruitment or overdistension occurs. Similarly, *E*_*drs*_ will evolve with time as recruitment is time dependent [27,28], disease state dependent [6,29] and MV dependent [28,30]. Arranging each breathing cycle’s *E*_*drs*_ curve such that it is bounded by the *E*_*drs*_ curve of the preceding breath and the subsequent breath leads to a three-dimensional, time-varying, breath-specific *E*_*drs*_ map. This method of visualisation gives new insight into how the breath-to-breath respiratory mechanics change with time over the course of treatment. In a similar manner, the corresponding change in airway pressure from PEEP to peak inspiratory pressure (PIP) is also displayed for each subject.

The dynamic elastance for each breath is calculated by dividing the numerator of Equation 5 by the volume vector. Therefore, at the very start of inspiration, when inspired volume is very small, *E*_*drs*_ approaches physiologically unrealistic values. Since overdistension is unlikely to occur at low volumes, the initial 20% of the inspiratory time for each breath is neglected for clarity. During this time, volume increases by approximately 0.04-0.06 L (less than 20% of the total inspired tidal volume) for each subject.

## Results

**Figure 1:**
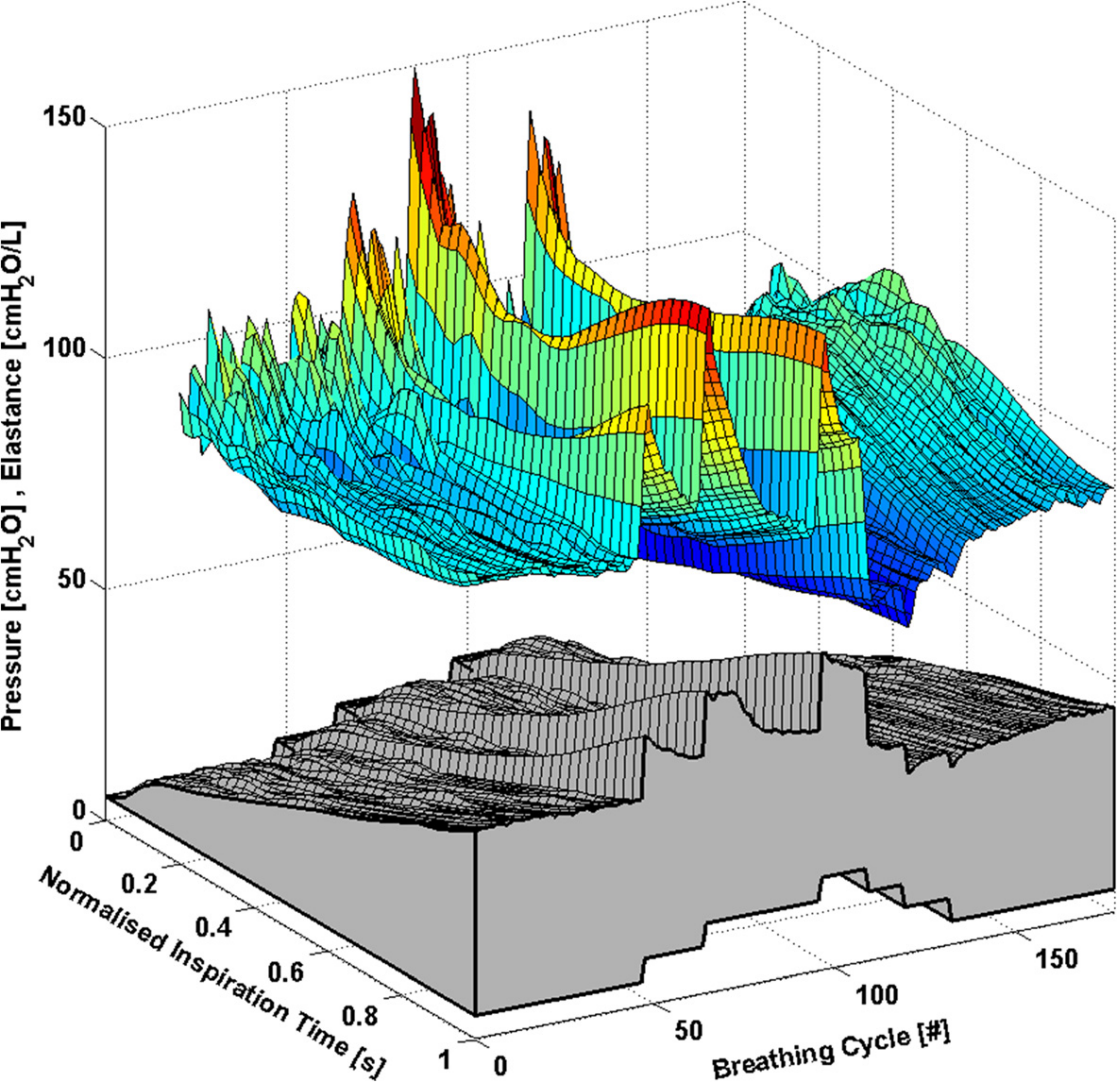
**Variation in *E***_***drs***_**across a normalised breath during a RM for Subject 1 (PaO**_**2**_**/FiO**_**2**_ **= 126.6 mmHg).** The change in airway pressure for each normalised breathing cycle is shown in grey.

**Figure 2:**
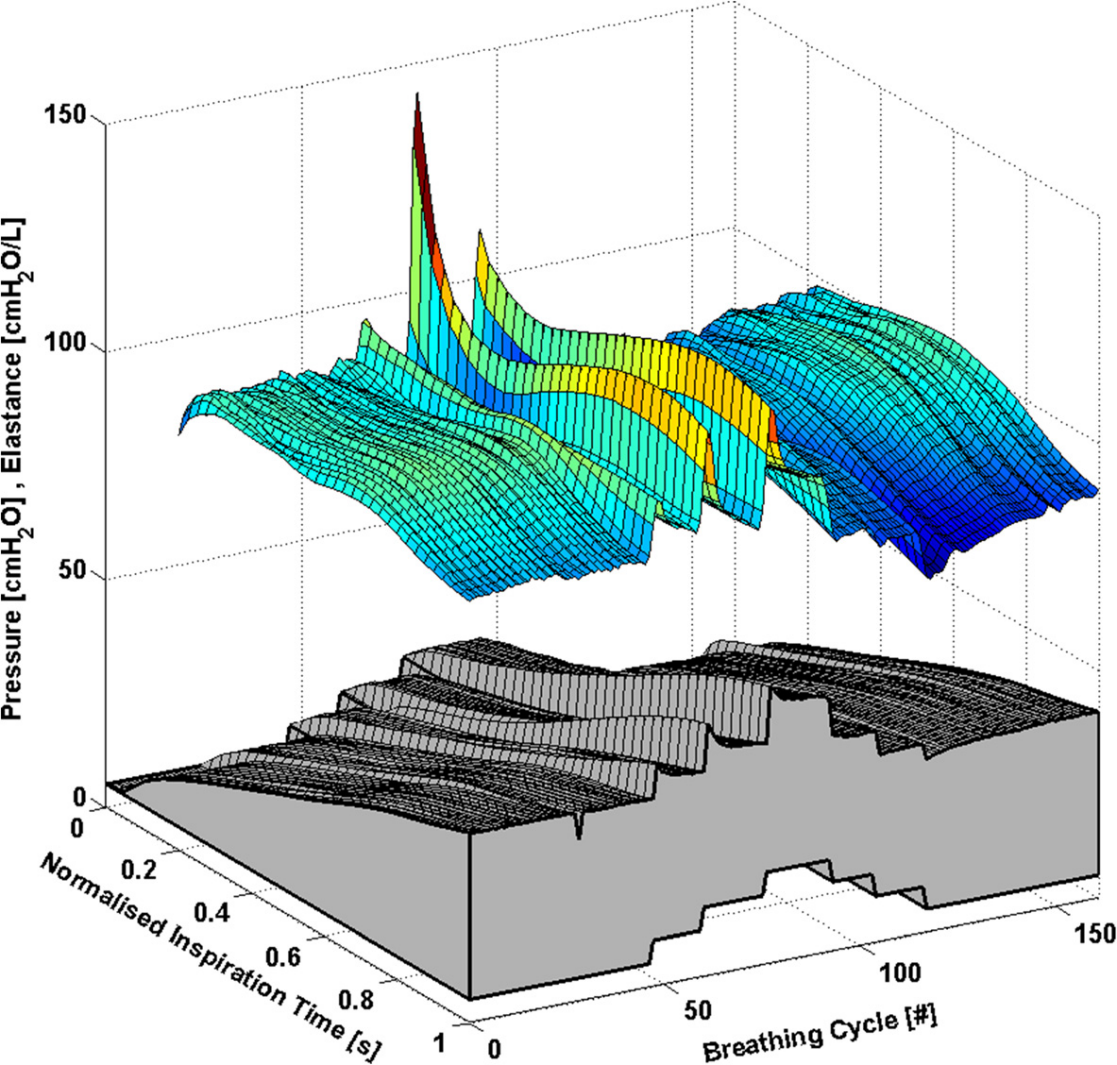
**Variation in *E***_***drs***_**across a normalised breath during a RM for Subject 2 (PaO**_**2**_**/FiO**_**2**_ **= 183.6 mmHg).** The change in airway pressure for each normalised breathing cycle is shown in grey.

**Figure 3:**
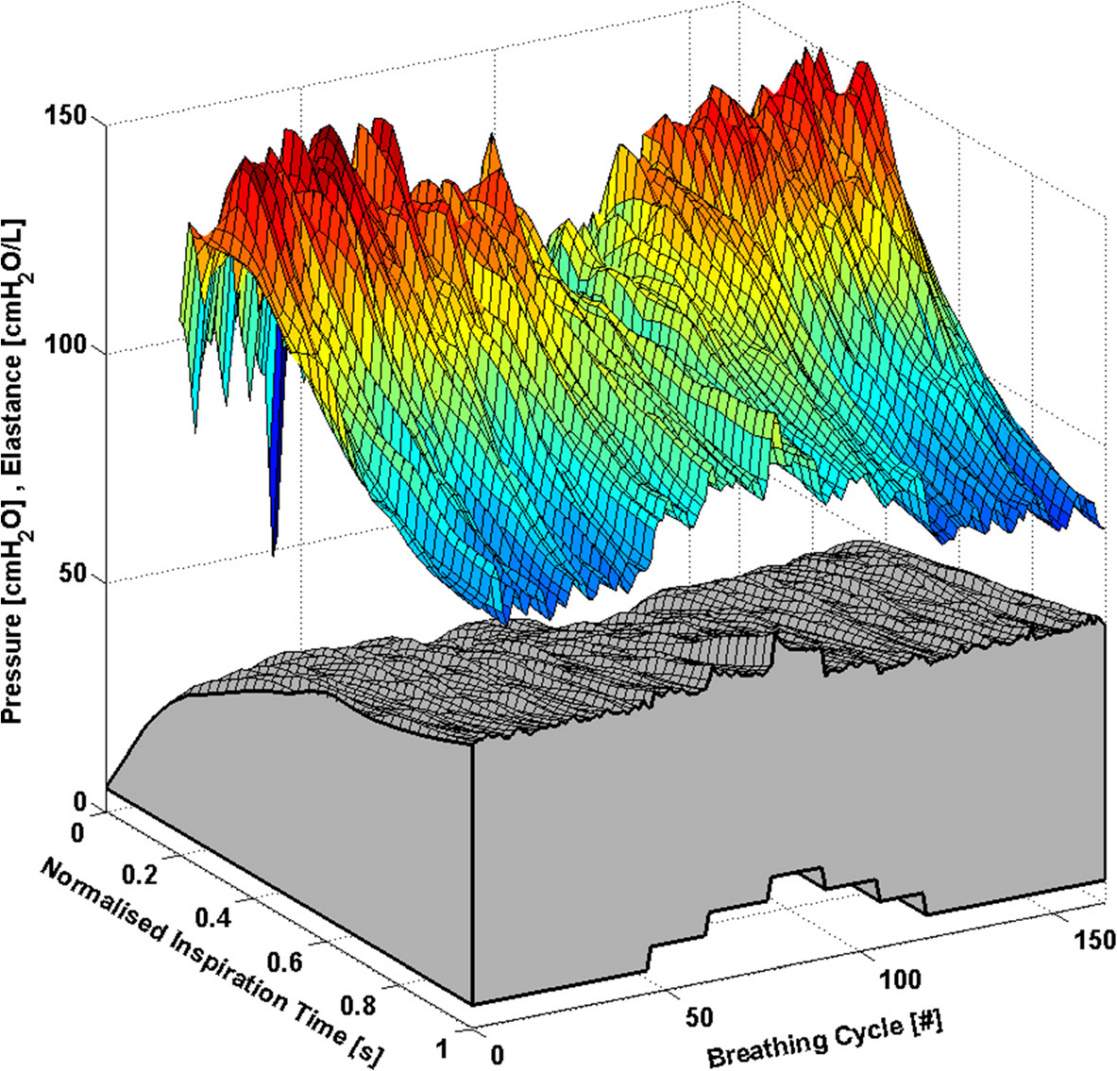
**Variation in *E***_***drs***_**across a normalised breath during a RM for Subject 3 (PaO**_**2**_**/FiO**_**2**_ **= 113.6 mmHg).** The change in airway pressure for each normalised breathing cycle is shown in grey.

**Figure 4:**
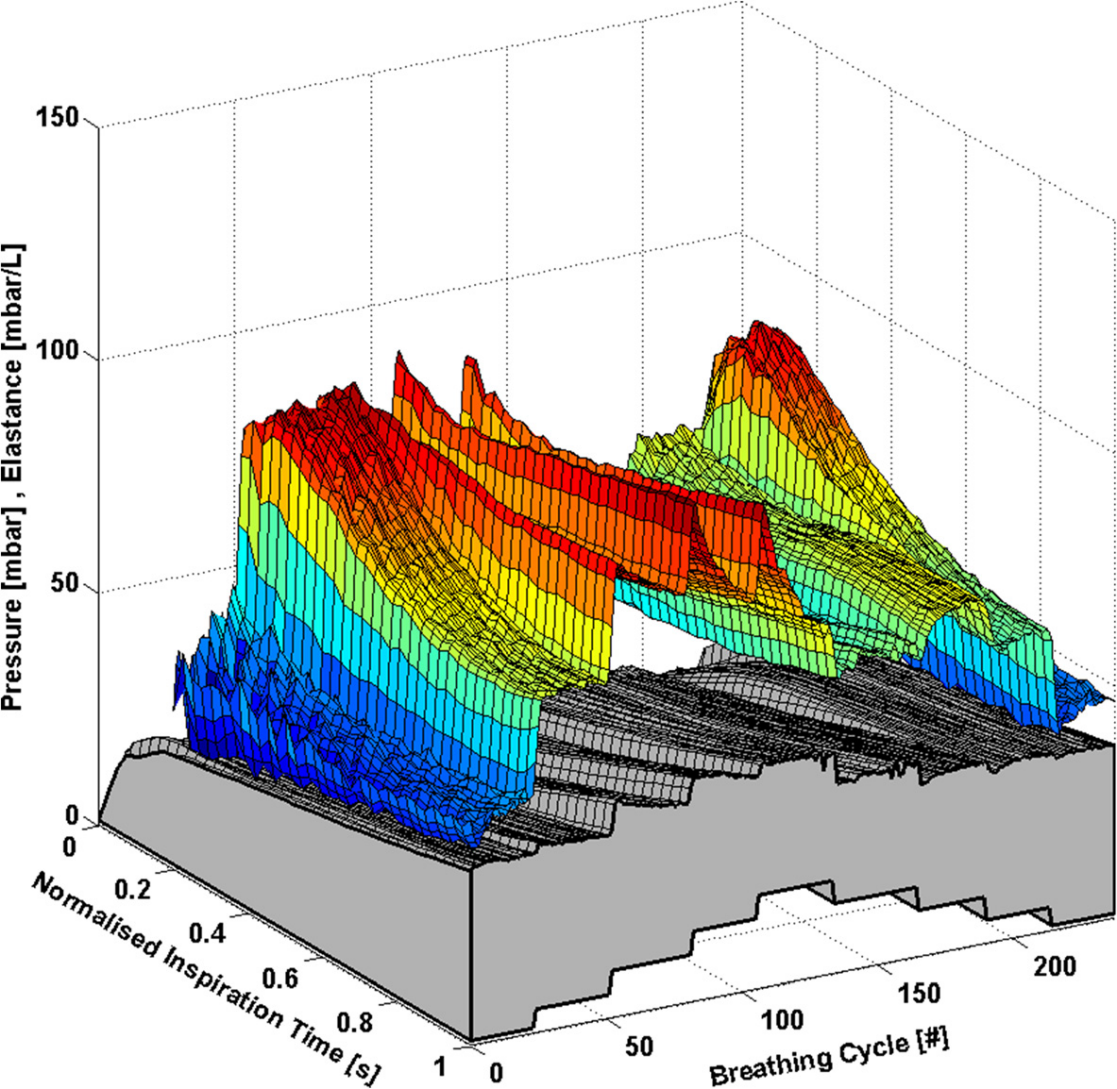
**Variation in *E***_***drs***_**across a normalised breath during a RM for Subject 4 (PaO**_**2**_**/FiO**_**2**_ **= 155.2 mmHg).** The change in airway pressure for each normalised breathing cycle is shown in grey.

**Figure 5:**
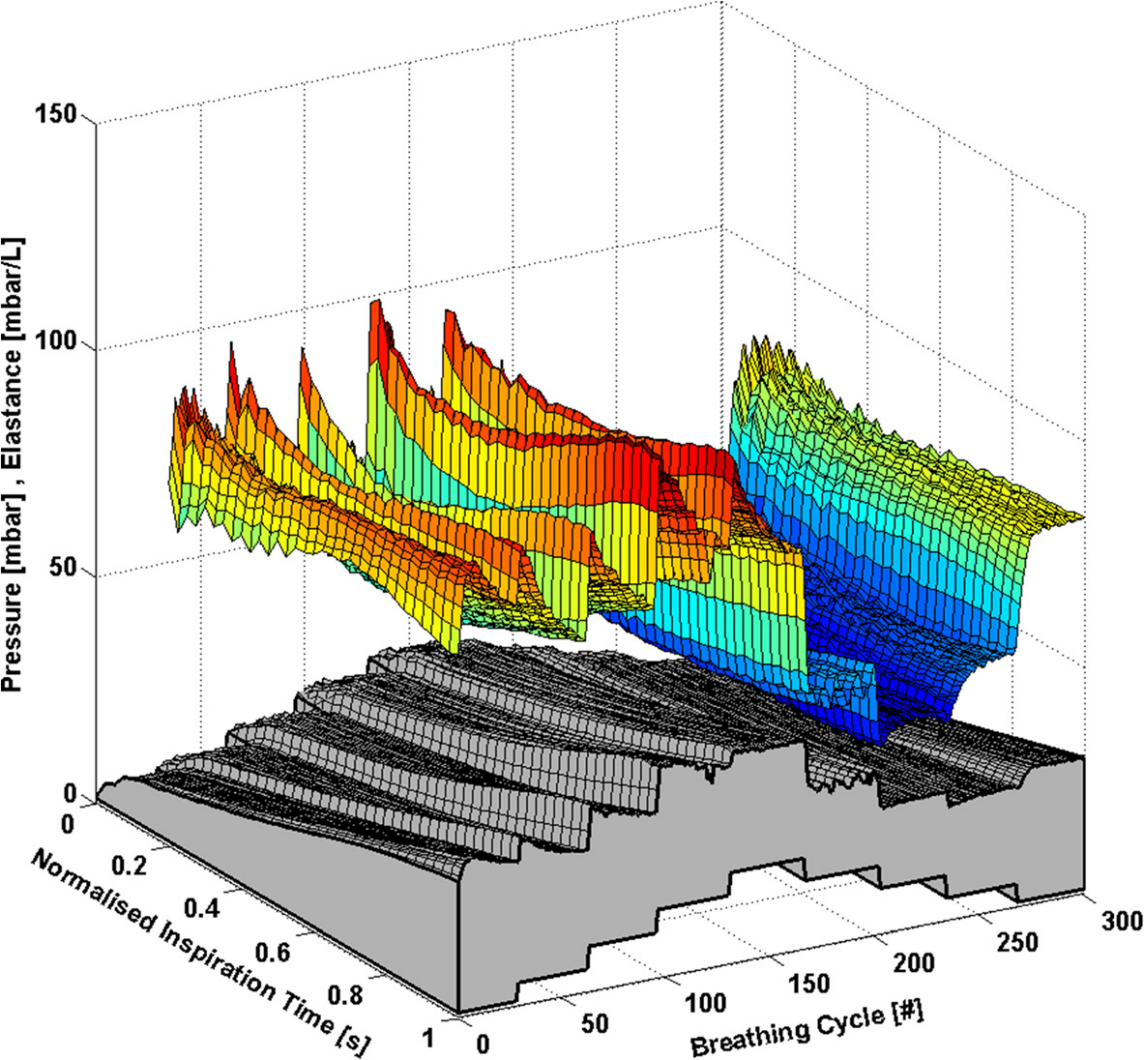
**Variation in *E***_***drs***_**across a normalised breath during a RM for Subject 5 (PaO**_**2**_**/FiO**_**2**_ **= 85.9 mmHg).** The change in airway pressure for each normalised breathing cycle is shown in grey.

**Figure 6:**
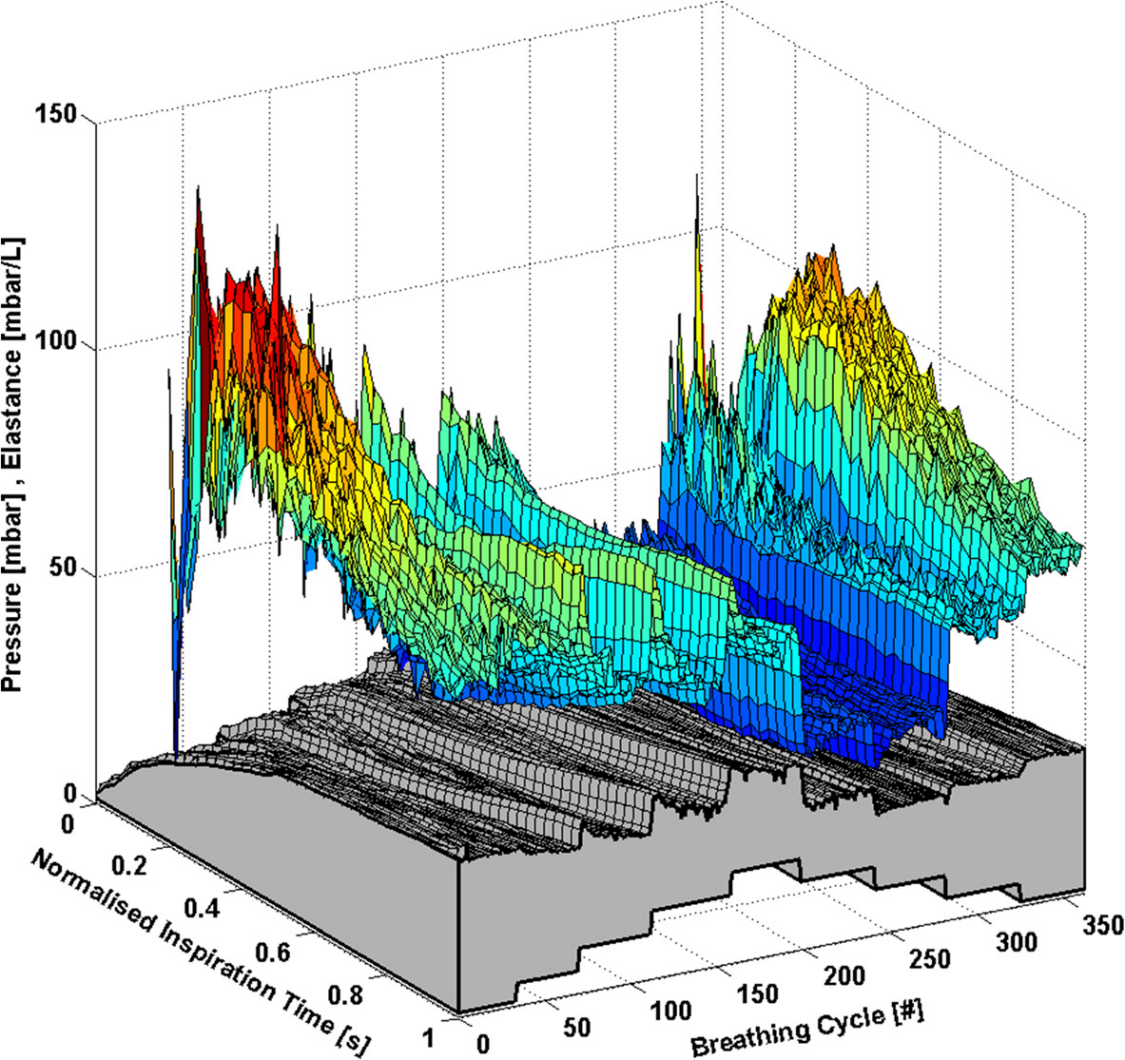
**Variation in *E***_***drs***_**across a normalised breath during a RM for Subject 6 (PaO**_**2**_**/FiO**_**2**_ **= 110.4 mmHg).** The change in airway pressure for each normalised breathing cycle is shown in grey.

Each subject has approximately 160 to 360 breathing cycles over the course of the RM. All breathing cycles are normalised to their total inspiratory time to provide clarity and to ensure consistency between breaths with different inspiratory times. Thus, each breath effectively begins at 0% and ends at 100% of the total inspiration time. The time-varying, breath-specific *E*_*drs*_ map of the RM for Subjects 1-6 are shown in Figures 1, 2, 3, 4, 5 and 6 respectively, where blue indicates low *E*_*drs*_ and red indicates high *E*_*drs*_. The corresponding airway pressure and PEEP are shown in grey. The PF ratio for each subject is stated in the corresponding figure caption. Each figure is also provided with a MATLAB figure in a compressed zip file to permit rotation. The *E*_*drs*_ and *R*_*rs*_ for each subject over the course of the RM is also shown within each MATLAB figure file (see Additional file 1). The top view of all *E*_*drs*_ maps are shown in Additional file 2.

## Discussion

### General observations

All subjects showed, to some degree, an increase in *E*_*drs*_ immediately following a PEEP step increase of 5 cmH_2_O or 5 mbar. Each successive breath had a reduced peak *E*_*drs*_ indicating the time-dependent nature of recruitment and/or the lung’s viscoelastic properties, which cause hysteresis [31,32]. More specifically, there is a period of adaptation following an increase in PEEP that sees higher average *E*_*drs*_, peak *E*_*drs*_ and PIP before the beneficial effect of lower *E*_*drs*_ is seen. Furthermore, the *E*_*drs*_ trajectory within a breath generally decreases during inspiration, suggesting in-breath recruitment. However, directly following a PEEP step increase, some subjects show a decreasing *E*_*drs*_ trajectory, followed by an increasing *E*_*drs*_ trajectory towards the end of inspiration. High elastance indicates serious potential for lung damage due to overstretching, and may not be captured by a single value of *E*_*rs*_[17,18]. Thus, as a result of this study, PEEP increments during a RM might be reduced to 1 cmH_2_O or 1 mbar, rather than increments of 5 cmH_2_O or 5 mbar, to avoid any damage due to the raised elastance in the early breaths and adaptation period following a PEEP increase. During a RM, smaller PEEP increments, each followed by a short period of stabilisation, may substantially reduce the peak of the *E*_*drs*_ spikes at the end of inspiration. However, it is important to note that the occurrence of lower respiratory elastance after stabilisation may also be a direct consequence of the initial high overdistension immediately following an increase in PEEP. This finding warrants further investigation where staircase recruitment is performed using smaller PEEP increments. Changes in ventilator pattern or mode to modify the *E*_*drs*_ trajectory also have potential to guide therapy.

The *E*_*drs*_ trend is significantly different between increasing and decreasing PEEP (using a non-parametric Wilcoxon rank sum test, p < 0.05 for each subject) where decreasing PEEP titration generally results in lower overall *E*_*drs*_. When PEEP increases, recruitment, as well as potential lung overstretching occurs. However, as PEEP is reduced, the lung remains compliant and *E*_*drs*_ drops to an overall minimum. Equally, this phenomenon is seen where the opening pressure of collapsed alveoli is higher than the closing pressure [29,33]. Considering increasing and decreasing PEEP separately, a local *E*_*drs*_ minimum generally occurs at the same PEEP level, suggesting that optimum PEEP can be selected either way. Recruitment is a function of PEEP and time [28,30], and, equally, the ARDS affected lung is prone to collapse due to the instability of affected lung units [6,29]. Assuming that the severity of ARDS does not change within a short period, respiratory elastance during increasing PEEP titration is expected to reduce as time progresses to achieve stability. In contrast, respiratory elastance will increase with time during decreased PEEP to achieve stability. Hence, the authors hypothesise that PEEP can be titrated to a minimum elastance either way, provided a stabilisation period is given at each PEEP level to obtain a true minimal elastance.

The time-varying *E*_*drs*_ map is a higher resolution metric of dynamic adaptation to PEEP than a single *E*_*rs*_ value. Selecting PEEP is a trade-off in minimising lung pressure and potential damage, versus maximising recruitment. Recruitment is a function of PEEP and time [28,30]. Therefore, true minimal *E*_*drs*_ can only be determined after a stabilisation period is provided at each PEEP level. Such a process could be readily automated and monitored in a ventilator. Setting PEEP at minimum elastance theoretically benefits ventilation by maximising recruitment, reducing work of breathing and minimising overdistension [12,14,15,34]. The PIP can be seen to follow the *E*_*drs*_ trend to some extent. However, it does not provide the same degree of resolution. In some cases PIP is seen to stabilise quickly or remain relatively constant following a change in PEEP, while *E*_*drs*_ continues to change significantly indicating the occurrence of significant lung dynamics not readily apparent from monitoring airway pressure alone. This result shows the greater sensitivity of using *E*_*drs*_ and that *E*_*drs*_ captures more relevant dynamics than airway pressure alone.

### Oleic acid ARDS models

#### Subject 1

The response of Subject 1 to PEEP titration is seen in Figure 1. The *E*_*drs*_ drops to an overall minimum at a PEEP of 15 cmH_2_O, suggesting that maintaining this level of PEEP provides the optimal trade-off between maximising recruitment and reducing the risk of lung damage [12].

#### Subject 2

The response of Subject 2 to PEEP titration is seen in Figure 2 and is similar to that of Subject 1. However, the magnitude of the *E*_*drs*_ response to PEEP is reduced. The *E*_*drs*_ drops to an overall minimum at a PEEP of 15 cmH_2_O, implying optimal PEEP.

#### Subject 3

In Subject 3, *E*_*drs*_ rises to a maximum near the beginning of each breathing cycle before rapidly decreasing as seen in Figure 3. However, this trend is less pronounced at high PEEP levels. It is observed that Subject 3 had a more severe level of ARDS (PaO_2_/FiO_2_ = 113.6 mmHg) compared to Subject 1 (PaO_2_/FiO_2_ = 126.6 mmHg) or Subject 2 (PaO_2_/FiO_2_ = 183.6 mmHg), possibly resulting in the substantially different subject-specific response to PEEP titration. The airway pressure curves show an initial rapid increase followed by a more gradual increase. It is possible that a different flow profile may eliminate the initial rapid pressure increase and reduce the rise in *E*_*drs*_. The most uniform elastance across a breath occurs at a PEEP of 15 cmH_2_O (at both increasing and decreasing PEEP), implying optimal PEEP.

### Lavage ARDS models

#### Subject 4

Respiratory elastance increases significantly in Subject 4 when PEEP is increased from 1 mbar to 5 mbar as seen in Figure 4. The lowest elastance is encountered either side of the RM at a PEEP of 1 mbar. However, *E*_*drs*_ reaches a local minimum at a PEEP of 15 mbar during decreasing PEEP. Thus, in this case, minimal elastance would suggest that the subject should be ventilated at 1 mbar rather than 15 mbar. However, it is important to note that atelectasis occurs in ARDS patients [35], and clinically, ARDS patients should be ventilated at higher PEEP [1,36,37]. Thus, this *E*_*drs*_ map outlines a potential drawback of ventilation considering only minimum elastance. Clinicians should thus consider an alternate PEEP value when an unrealistically low PEEP is recommended by elastance.

#### Subject 5

The response of Subject 5 to PEEP titration is seen in Figure 5. The *E*_*drs*_ drops to an overall minimum at a PEEP of 10 mbar, implying optimal PEEP.

#### Subject 6

Unlike Subjects 4 and 5, the RM performed on Subject 6 was performed during an open chest surgery, thereby neglecting the effect of *E*_*cw*_ in Equation 2 and effectively capturing *E*_*lung*_ directly. It was found that more noise was present in this trial when compared to closed chest ventilation performed on Subjects 1–5. The noise present in this data indicates that the chest wall may provide some form of damping to high frequency physiological or mechanical effects. It was observed that minimum elastance occurs at a decreasing PEEP of 10 mbar, as shown in Figure 6.

### Limitations

The single compartment lung model used to derive *E*_*drs*_ does not capture some specific physiological aspects, such as cardiogenic oscillations or regional differences in mechanical properties [21]. Furthermore, the effects of non-linear flow or variations in airway resistance during a breath are also neglected [21]. The determination of *E*_*drs*_ accommodates whatever resistance value is chosen, such that the model perfectly fits the available pressure data. Hence, the assumption of constant resistance throughout a breath significantly impacts on the trends of *E*_*drs*_. There is evidence to suggest that in some cases respiratory resistance can vary within a breath [23]. However, the effect of the resistive term is mathematically limited in its impact [17]. Since this analysis is predominantly based on the comparison of trends across PEEP values, where each subject is thus their own reference, the best validation is the ability to track clinically expected trends as shown here.

It is important to note that both ARDS animal models were different in many aspects and do not allow for a statistically significant comparison. More importantly, it was not able to fully justify PEEP optimisation based solely on minimal elastance. However, the main outcome of this research is that mapping of time-varying respiratory elastance of mechanically ventilated ARDS subjects can be monitored to provide a high resolution metric to describe disease state and physiological changes in response to PEEP. This outcome shows the robustness of both the model and the method of visualisation for application in the ICU. However, more inter-patient variability is present in patients admitted to the ICU. Thus, application of this monitoring technique warrants further investigation in both human and animal studies.

Selecting patient-specific optimal PEEP remains widely debatable with little consensus [36,37]. This study primarily provides a means to visualise respiratory system elastance continuously, thus allowing PEEP to be titrated to minimal elastance [12,14,15], and it was suggested that it can be done using either incremental or decremental phase of a staircase RM. However, this suggestion is limited to the protocol and data available. If only the incremental phase of the RM is available, PEEP titration can be performed during incremental stage, or vice-versa. If both incremental and decremental phase of the staircase RM are available, PEEP should be titrated during decreasing PEEP, as the incremental PEEP functions to recruit the collapsed lung [38,39].

A further limitation is that the findings of this research are solely based on observation of the *E*_*drs*_ map. The findings require further investigation together with additional imaging and monitoring tools such as in-vivo microscopy, computer tomography and/or electrical impedance tomography for validation. However, high resolution imaging technology is currently limited to regional investigation and clinically impractical for full and continuous monitoring [40-42]. Thus, the findings of this research are limited to comparisons with existing literature.

## Conclusion

Visualisation of the dynamic respiratory elastance provides significantly more insight into dynamic lung behaviour than can be provided by a single value of *E*_*rs*_. Simultaneous monitoring of respiratory elastance across a breath and during a RM provides a new clinical perspective to guide therapy and provides unique subject-specific insight into the heterogeneous response to PEEP. The model is limited to a constant respiratory resistance throughout inspiration which may not be valid in some cases. However, trends match clinical expectation and the results highlight both the subject-specificity of the model, as well as significant inter-subject variability. Overall, further research is warranted to confirm the clinical potential of using this method in ARDS patients admitted to the ICU.

## Abbreviations

ARDS: Acute respiratory distress syndrome; ICU: Intensive care unit; MV: Mechanical ventilation; PEEP: Positive end expiratory pressure; WOB: Work of breathing; PF: PaO_2_/FiO_2_; ABG: Arterial blood gas; RM: Recruitment manoeuvre; PIP: Peak inspiratory pressure.

## Competing interests

The authors declare that they have no competing interests.

## Authors’ contribution

EJD assisted in the development of the *E*_*drs*_ model, generated the *E*_*drs*_ maps, and drafted the manuscript. YSC participated in the implementation of the clinical trials, assisted in the development of the *E*_*drs*_ model, and helped to draft the manuscript. CP participated in the implementation of the clinical trials. GMS participated in the implementation and coordination of the study. BL and NJ participated in the implementation of the clinical trials. JGC participated in the implementation and coordination of the study, and helped to draft the manuscript. TD participated in the implementation and coordination of the study, implementation of the clinical trials, and helped to draft the manuscript. All authors read and approved the final manuscript.

## Pre-publication history

The pre-publication history for this paper can be accessed here:

http://www.biomedcentral.com/1471-2466/14/33/prepub

## Supplementary Material

Additional file 1 Subject 1 - Oleic acid ARDS. Subject 2 - Oleic acid ARDS. Subject 3 - Oleic acid ARDS. Subject 4 - Lavage ARDS. Subject 5 - Lavage ARDS. Subject 6 - Lavage ARDS. Click here for file

Additional file 2 **Top view of *E***_***drs***_**map for each subject.** Click here for file
