# Supplementary material for: Visualisation of time-varying respiratory system elastance in experimental ARDS animal models
Source: BMC Pulm Med. 2014 Mar 2;14:33. doi: 10.1186/1471-2466-14-33 (PMC4016000; doi:10.1186/1471-2466-14-33)
Supplement: Additional file 2 — Top view of E drs map for each subject. [file 1471-2466-14-33-S2.docx]

*Additional File: Top View of E_drs_ Map for Each Subject*

| *Subject 1 (Oleic Acid)* | *Subject 4 (Lavage)* |
| --- | --- |
| *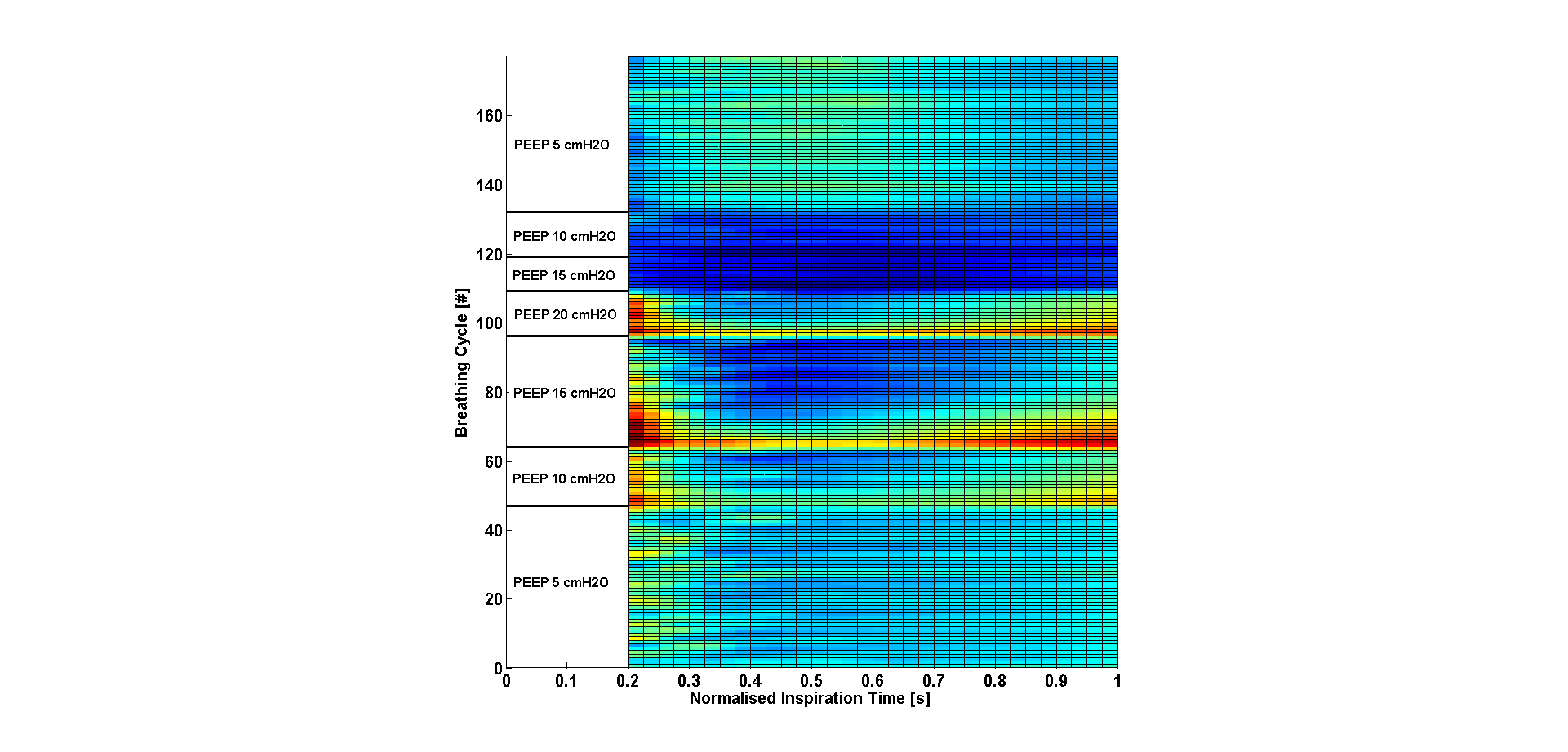* | *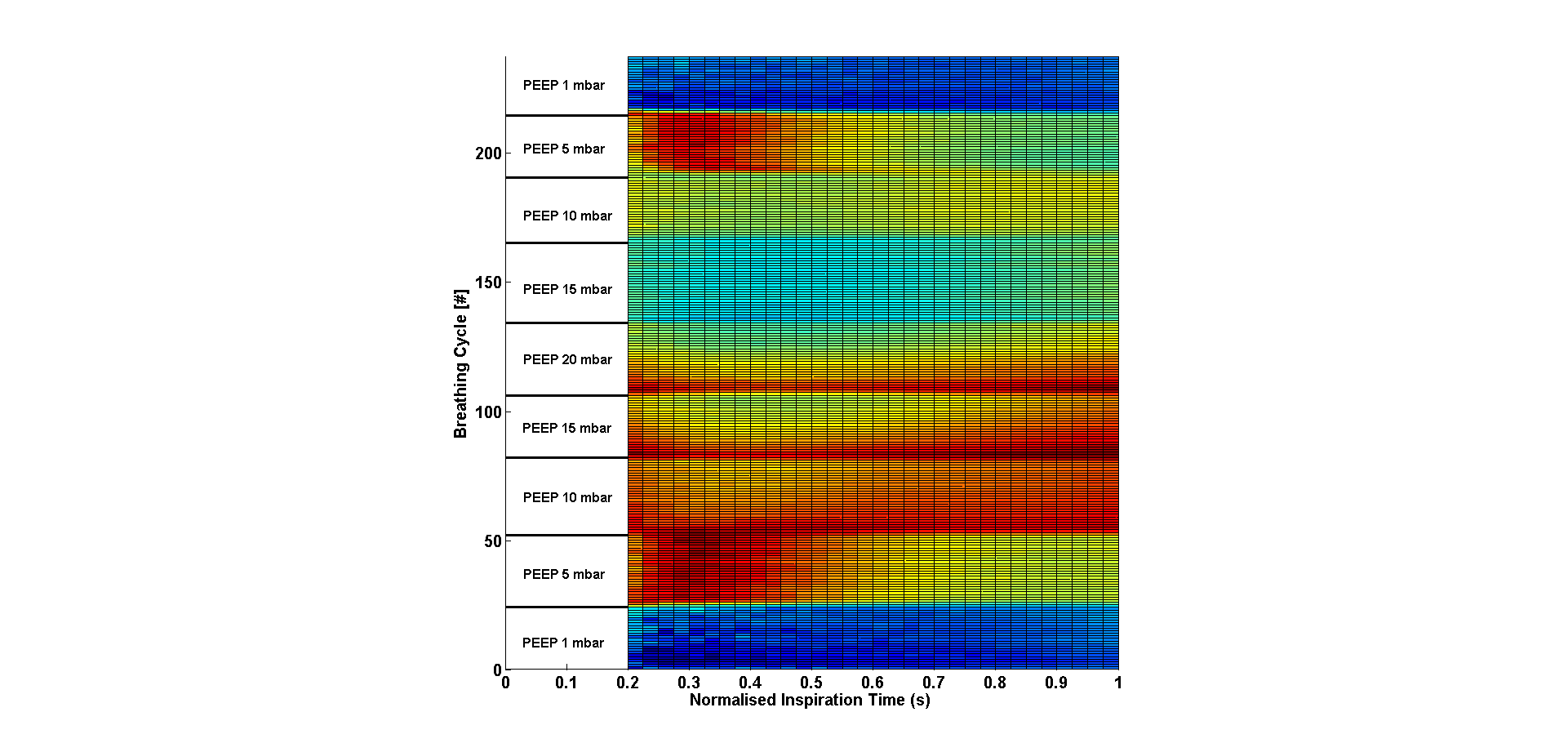* |
| *Subject 2 (Oleic Acid)* | *Subject 5 (Lavage)* |
| *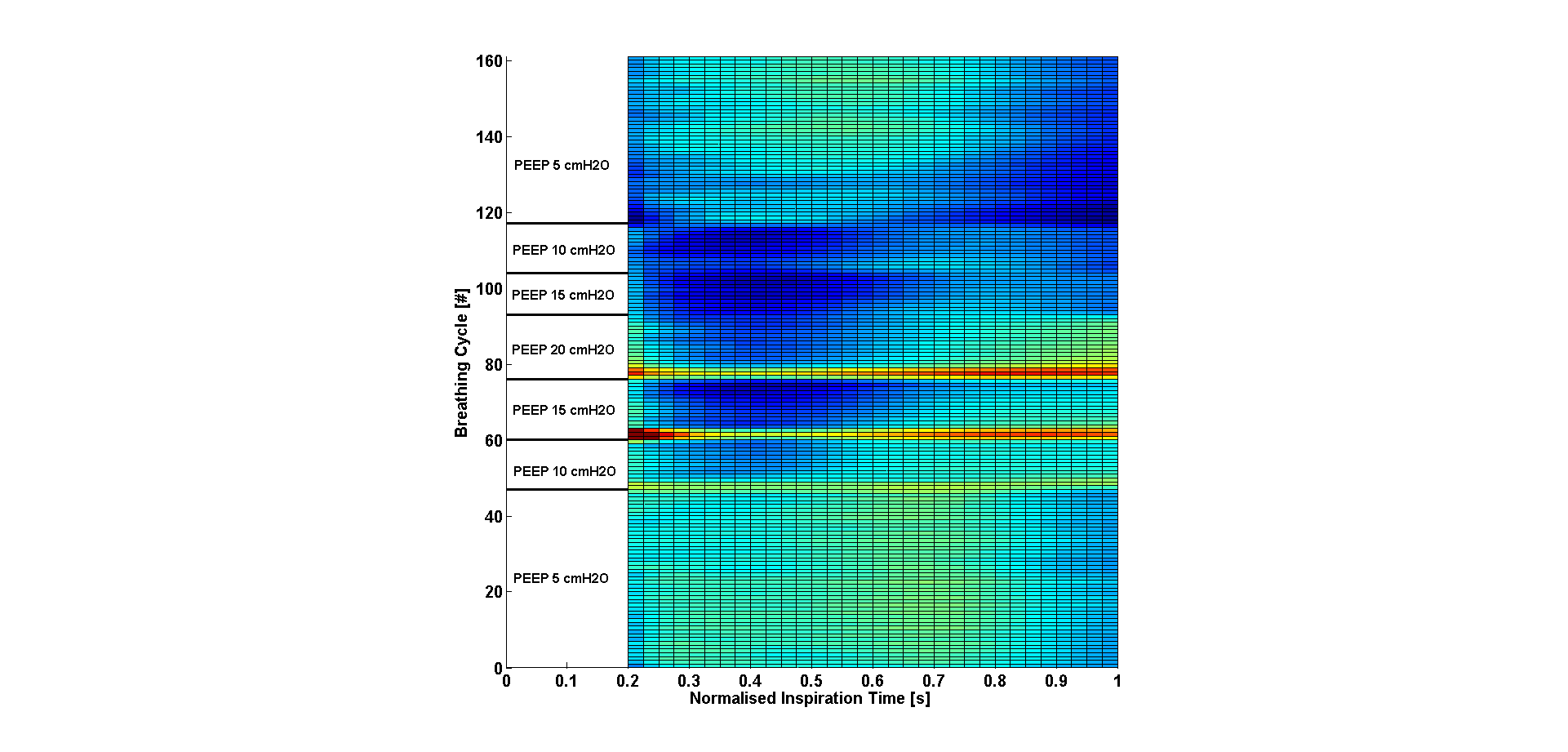* | *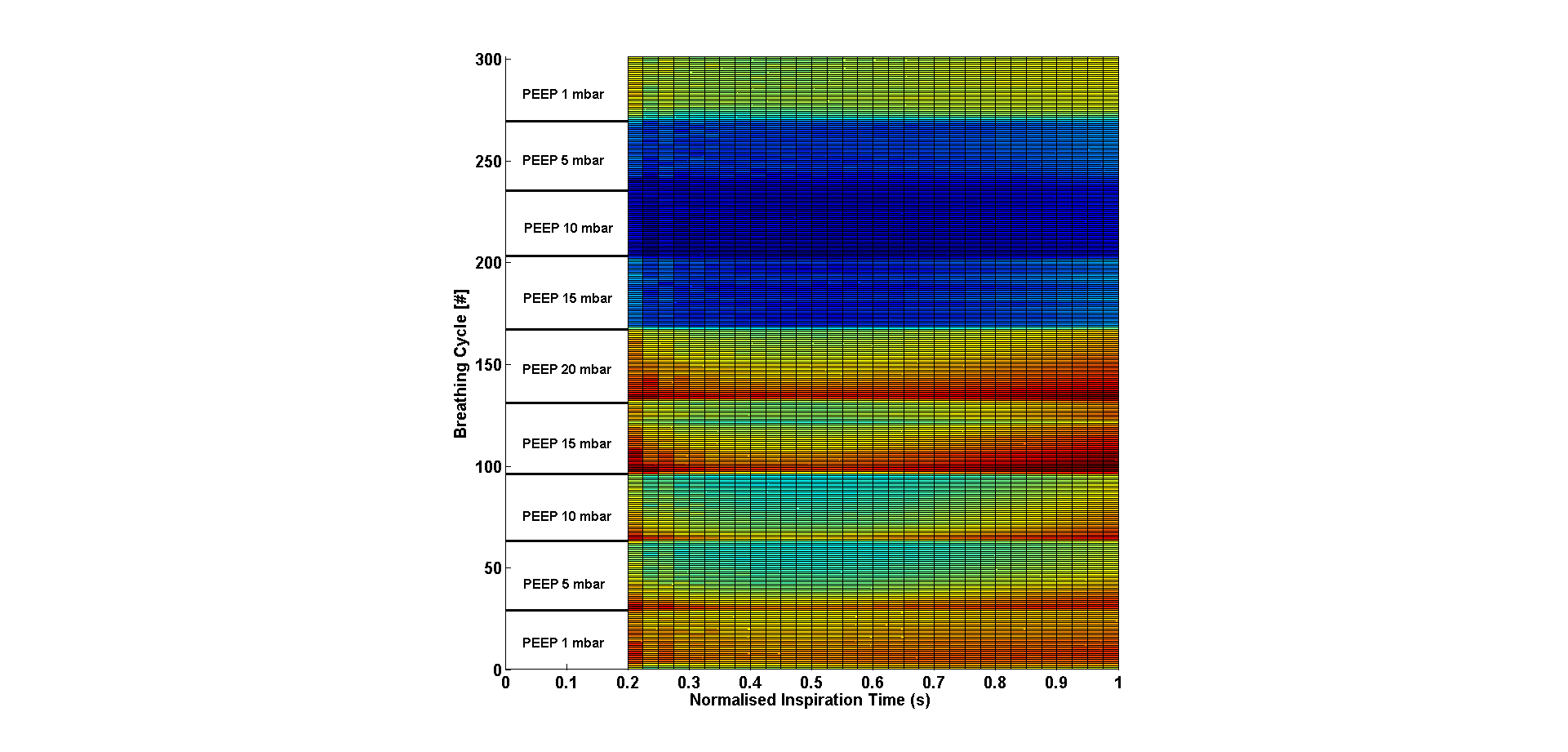* |
| *Subject 3 (Oleic Acid)* | *Subject 6 (Lavage)* |
| *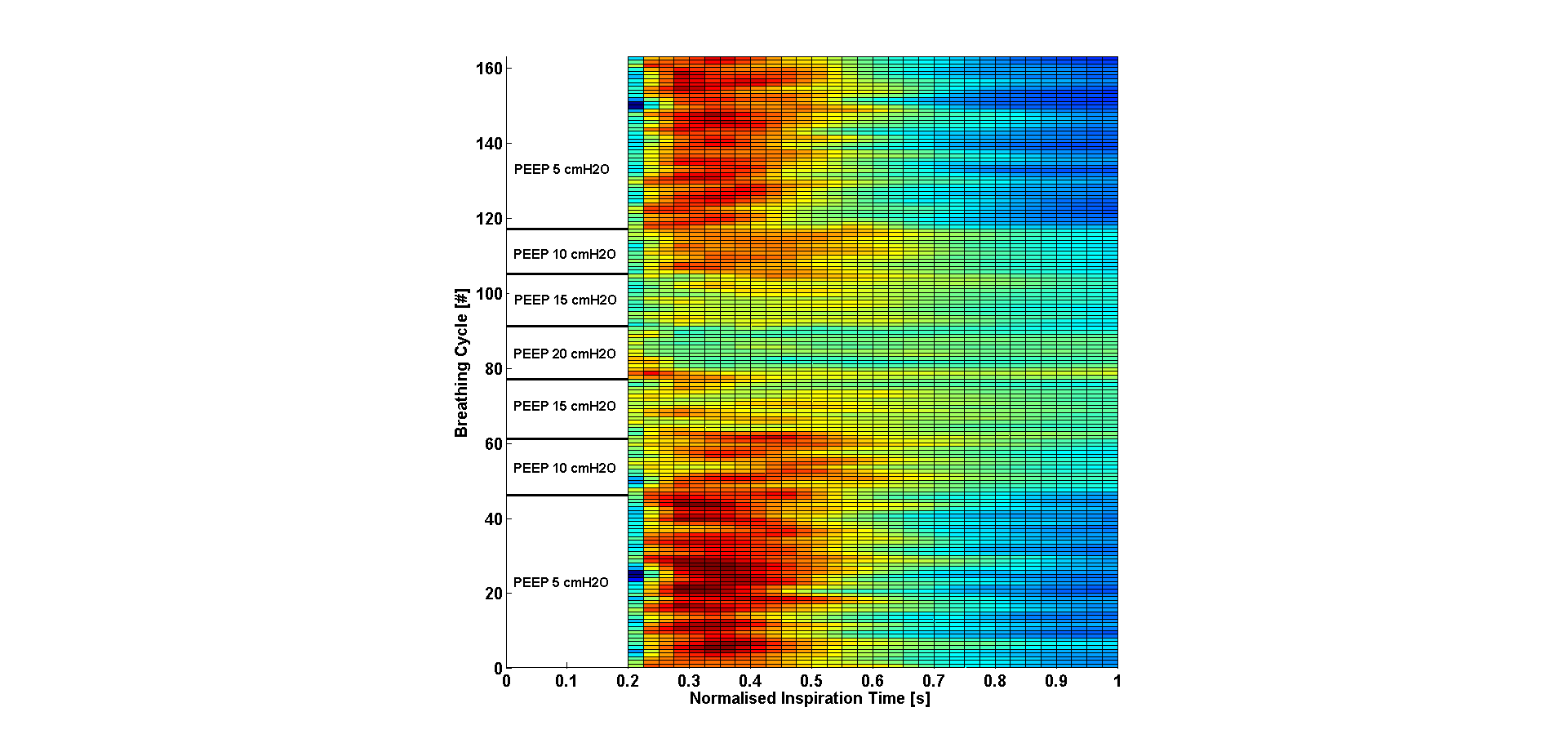* | *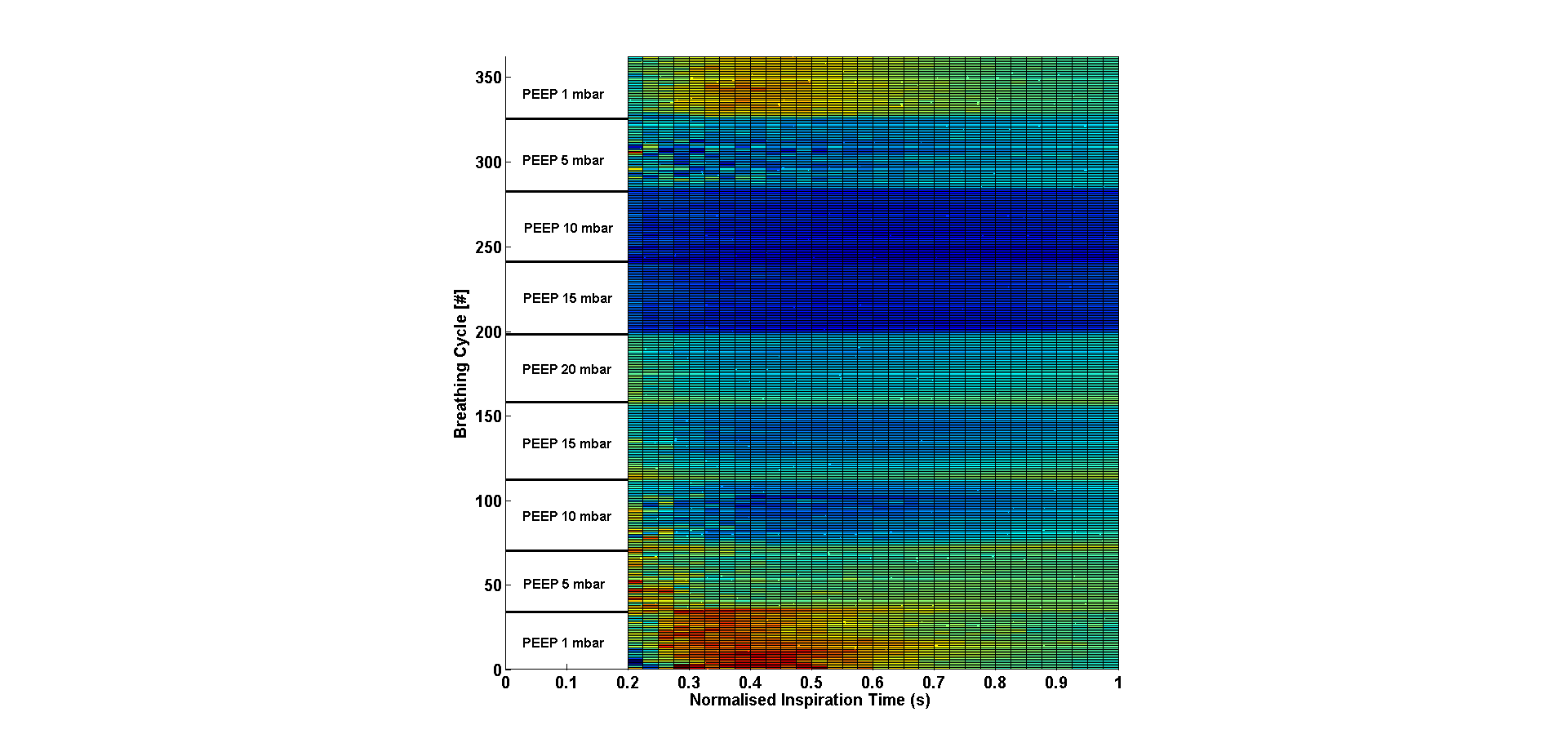* |
